# Surgical factors related to dental implant failure: A cross-sectional multicentre study on 1,308 dental implants

**DOI:** 10.4317/medoral.28046

**Published:** 2026-04-19

**Authors:** Ángel Orión Salgado-Peralvo, Antonio Murillo-Rodríguez, Francisco Javier Díaz-Prada, José Luis Megía-Martín-Peñasco, Pablo Galarza-Estebaranz, Óscar Lado-Baleato, Mario Pérez-Sayáns

**Affiliations:** 1 Department of Surgery and Medical-Surgical Specialties, Faculty of Medicine and Dentistry, University of Santiago de Compostela, 15782 Santiago de Compostela, Spain; 2 Health Research Institute of Santiago de Compostela (IDIS), ORALRES Group, Santiago de Compostela, Spain; 3 Private practitioner; 4 Department of Surgery, Radiology and Medical physics, University of the Basque Country, 48940 Biscay, Spain; 5 Research Methodology Group Clinical Research Support Platform (ISCIII); 6 Instituto de los materiales de Santiago de Compostela (iMATUS), Spain

## Abstract

**Background:**

Implant failure is equivalent to the loss of the implant, which entails biological and economic costs and reduces patient's trust in healthcare professionals. The objective of this study was to estimate the cumulative failure rate of 1,308 implants and identify patient-related and surgical factors associated with implant failure.

**Material and Methods:**

Data for this retrospective multicentre study were extracted from six private clinics between June 2006 and December 2022. Patient-related factors, surgical techniques, and implant-related variables were evaluated. Multivariate mixed Cox proportional hazards models and Lasso regularisation were employed to identify predictors of implant failure.

**Results:**

A total of 1,308 implants (510 patients) were inserted, with a failure rate of 2.4% over a mean follow-up period of 38.31±32.54 months. The selected mixed Cox regression models showed that three variables were independently associated with implant failure: Postoperative implant infection (Hazard Ratio [HR]=6.6 [95% CI: 1.8-23.9]; p=0.004), previous implant failure (HR=8.3 [95% CI: 2.6-26.6]; p&lt;0.001), and heavy smoking (&gt;20 cigarettes per day) (HR=99.3 [95% CI: 30.7-321.3]; p&lt;0.001).

**Conclusions:**

With the limitations of the present study, the cumulative implant failure rate was low (2.4%). Nevertheless, strict control of smoking habits and infection risk factors is essential, since implant replacement after failure constitutes and independent risk factor for subsequent failure.

## Introduction

Dental implants are considered the most predictable therapeutic option for the partial or complete replacement of missing teeth, with reported survival rates of approximately 95% in both pristine and regenerated bone (1). Achieving and maintaining osseointegration is a sine qua non condition for successful implant therapy. Osseointegration was originally defined as "a direct structural and functional connection between ordered living bone and the surface of a load-carrying implant" (2).

Implant failure has been defined in several ways in the scientific literature. Chrcanovic et al. (3) described "implant failure" as the clinical signs and symptoms leading to explantation, whereby 'failure' is equivalent to implant loss. Criteria for determining implant viability have been widely reported (4 , 5). The International Congress of Oral Implantologists Pisa Consensus Conference (4) defined "implant survival" as the persistence of a dental implant in the oral cavity in the absence of mobility, pain on function, or bone loss greater than 50% of the implant length. Furthermore, Albrektsson et al. (6) proposed that implant success requires the absence of mobility, absence of radiolucency at the implant-bone interface, marginal bone loss (MBL) of less than 0.2mm per year after the first year of functional loading, and absence of persistent pain, discomfort, or infection.

Overall implant failure rates have been reported to range between 0.7 and 3.8%. Implant failures are commonly classified as early or late, depending on whether they occur before or after prosthetic loading (7). This distinction is clinically relevant because different biological and mechanical factors are involved. Early failures are generally related to impaired osseointegration due to local and/or systemic factors and are usually associated with limited peri-implant bone loss, accounting for approximately 5% of all failures (3). In contrast, late failures are more frequently associated with biological factors (e.g., biofilm-related peri-implantitis) and mechanical factors (e.g., fatigue, overload, or corrosion), leading to progressive bone reabsorption and affecting the majority of implants that initially achieve osseointegration (8).

The primary objective of this retrospective study was to determine the cumulative failure rate of 1,308 dental implants. The secondary objective was to evaluate patient-related, surgical, and implant-related factors associated with implant failure and survival, with the aim of reducing future failure rates.

## Material and Methods

Study design and ethical approval

This retrospective multicentre study was conducted in six private dental clinics in Spain between June 2006 and December 2022 and was reported in accordance with the STROBE guidelines. All procedures were performed in accordance with the ethical standards of the institutional and/or national research committee and with the Declaration of Helsinki and its later amendments. The research protocol was approved by the Ethics Committee of the CEIC Hospital Clínico San Carlos (Madrid, Spain) (Registration No. 23/741-O_P).

Participants

All participants received clear information regarding the nature and objectives of the study and provided written informed consent prior to inclusion.

Eligible participants were men and women aged 18 years who were partially or fully edentulous, presented with a plaque index 20%, and had been rehabilitated with Galimplant dental implants (Sarria, Lugo, Spain), with a minimum follow-up of 12 months after prosthetic loading. Only patients classified as ASA I or II were included.

Excluded criteria comprised severe systemic disease (ASA III or IV), untreated or uncontrolled periodontal disease, coagulation disorders, bruxism, pregnancy or breastfeeding, disorders affecting bone metabolism, inadequate implant maintenance, ongoing or recent (&lt;2 years) head and neck radiotherapy or chemotherapy, immunosuppression, and alcohol or drug abuse.

Sample size calculation

Based on recent data, approximately 20% of the Spanish population aged 25-79 years has at least one dental implant. According to the Spanish National Statistics Institute, the total population in the provinces where data were collected (Pontevedra, León, Vizcaya, and Ciudad Real) was 2,181,256 inhabitants in 2022, of whom an estimated 436,251 carried dental implants. Assuming a statistical power of 90%, a confidence interval (CI) of 95%, and a margin of error of 5%, a minimum sample size of 385 patients was required to detect statistically significant differences.

Dental implant characteristics

A single implant system (Galimplant, Sarria, Lugo, Spain) was used. The implants feature a Nanoblast plus® surface composed of 99.9% TiO2, obtained through coarse-grained sandblasting followed by triple acid etching, resulting in a surface roughness of Sa=1.7 m. The system includes implants with hexagonal conical internal connection and universal external connection.

Data extraction

Each participating centre retrospectively collected patient data in a pseudo-anonymised Excel database specifically designed for this study. Recorded variables included patient-related factors, surgical techniques, and implant-related characteristics.

Statistical analyses

For each implant, survival time was calculated from the date of placement to the date of failure. Implants that did not fail by the end of the observation period and those lost to follow-up were treated as censored observations.

Associations between patient- and implant-related variables and the risk of implant failure were analysed using the multivariate Cox proportional hazards models. Mixed-effects models with random intercepts were applied to account for clustering of multiple implants within the same patient.

Lasso regularisation with cross-validation was used for variable selection to minimise overfitting. Selected variables were subsequently included in the multivariate mixed Cox regression model. Variables with p-values &gt;0.05 were removed from the final model. Proportional hazards assumptions were verified, and time-dependent area under the curve (AUC) values were calculated to assess model discrimination.

All analyses were performed using R statistical software (R Core Team, 2024). The mixed Cox proportional hazards model was fitted using the coxme package (version 2.2-22). Model performance was evaluated using the timeROC package.

## Results

Descriptive data

A total of 510 patients were included, 289 women (56.67%) and 221 men (43.33%), with a mean age of 59.98±11.34 years (range: 21-95 years). A total of 1,308 implants were analysed (722 in women [55.2%] and 586 in men [44.8%]). The implant survival rate was 97.6% over a mean follow-up period of 38.31±32.54 months. Most implants were placed in non-smokers (71.1%). Among smokers, the most frequent consumption was 11-20 cigarettes/day (19.6%). The mean time from implant placement to prosthetic loading was 7.10±8.34 months, with the conventional loading protocol being the most commonly used (92.9%) (Table 1).

Table 1Most implants had an internal connection compared with external connection implants (99.4% vs. 0.6%). More than half of the implants were 4mm in diameter (53.8%), followed by 3.5mm (31.1%). The most frequently used lengths were 10mm (41.4%) and 8mm (25.8%), followed by 12mm (25.1%).

Overall, 82.3% of the implants were inserted without simultaneous bone regeneration, while 3.2% required prior regeneration. The most frequent regenerative procedures were immediate implant placement with particulate bone graft filling (9.0%) and guided bone regeneration (GBR) (5.3%).

Most implants were located in the posterior region (83.6%), with a similar distribution between the maxilla (54.6%) and mandible (45.4%). Only 2.4% of the implants were placed to replace a previously failed implant. In approximately two-thirds of the cases, the antagonist dentition was natural (66.6%).

Preventive antibiotic therapy (PAT) was administered in 99.6% of the surgeries. The postoperative infection rate was 2.5% (32 implants), with a mean onset of 3.15±10.45 days (range: 0.03 to 58.6 days)

Dental implant failure

**Figure 1:**
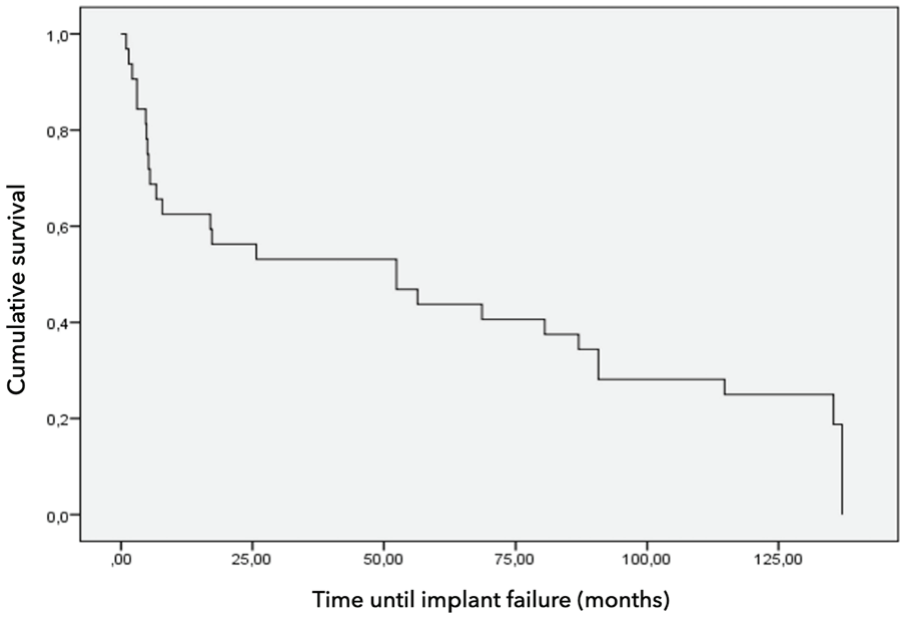
Kaplan-Meier global survival curve.

The implant failure rate was 2.4%, with failures occurring at a mean of 59.29±9.81 months (range: 40.06-78.53 months) (Figure 1). Implants failed significantly more frequently in smokers than in non-smokers (4.4% vs. 1.8%; p=0.022), particularly in heavy smokers (&gt;20 cigarettes/day; 47.1%; p&lt;0.001). However, among smokers, failures occurred later, resulting in longer survival times compared with non-smokers (85.07 vs. 36.54 months; p=0.003).

1Regarding implant characteristics, significantly higher failure rates were observed in external connection implants compared with internal connection implants (37.5% vs. 2.2%; p&lt;0.001), in implants placed in the maxilla compared with the mandible (9% vs. 1.5%; p=0.047), and in the anterior region compared with the posterior region (22% vs. 2%; p=0.022). Implant failure was strongly associated with previous surgical site infection (21.9%; p&lt;0.001) and with replacement of a previously failed implant (19.4%; p&lt;0.001) (Table 2). Replacement implants showed significantly shorter survival than implants placed for the first time (23.57 months vs. 67.54 months; p=0.025) (Table 3).

**Figure 2:**
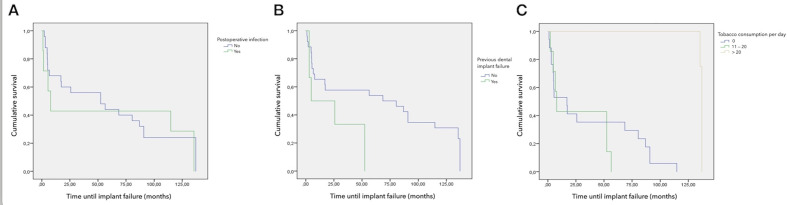
Kaplan-Meier survival curves for (a) postoperative infection, (b) previous dental implant failure and (c) heavy smoking (&gt;20 cigarettes per day).

Table 2Table 3The selected mixed Cox regression models identified three variables independently associated with implant failure: Postoperative implant infection, previous implant failure, and heavy smoking (&gt;20 cigarettes per day). The effects of these variables are expressed as Hazard Ratios (HR) with 95% CI. P-values are reported for each variable. In this regard, postoperative infection increased the risk of failure by 6.6-fold (95% CI: 1.8-23.9; p=0.004) (Figure 2A). Previous failure increased the risk by 8.3-fold (95% CI: 2.6-26.6; p&lt;0.001) (Figure 2B). Heavy smoking showed a very strong association, increasing the risk of failure by 99.3-fold (95% CI: 30.7-321.3; p&lt;0.001) (Figure 2C).

**Figure 3:**
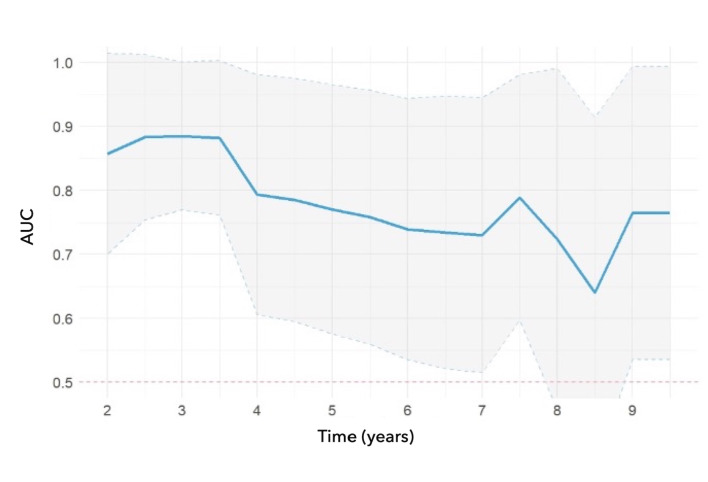
Time-dependent area under the curve (AUC) for the multivariate Cox proportional hazard regression model.

2The final model showed a C-index of 0.863, indicating a good discriminative ability. Figure 3 illustrates the time-dependent AUC over a 9-year period. The AUC remained close to 0.9 during the first four years after the implant placement and gradually declined thereafter, reaching a minimum around year 8, followed by a slight recovery. Wider confidence intervals at later time points indicated increased uncertainty in these estimates.

3

## Discussion

The present retrospective study documented 32 implant failures among 510 patients in whom 1,308 implants were placed over a mean follow-up period of 3.19 years, resulting in a cumulative survival rate of 97.6%. These results are consistent with those reported in a systematic review describing a 96.4% survival rate after 10 years of follow-up (1).

The evidence regarding age and sex as risk factors for implant failure remains controversial. In the present study, a similar failure rate was observed between men and women (2.9% vs. 2.1%; p=0.338), in agreement with previous reports (9 , 10). With respect to age, most failures occurred in patients aged 40 to 65 years (81.3%), followed by those &gt;65 years (15.6%), whereas patients &lt;40 years accounted for only 3.1% of failures (p=0.057). This finding is consistent with a recent study reporting a 4-5-fold higher risk of failure in patients older than 40 years. However, multiple regression analyses have shown that patients older than 51 years usually receive a higher number of implants, which has been associated with and increased risk of failure (Odds Ratio [OR]=1.30; p&lt;0.001), suggesting that the effect may be related more to implant burden than to age per se (11).

Regarding implant location, several studies, including the present one, have reported a higher rate of late failure in implants placed in the maxilla, with HR ranging from 2.59 (p&lt;0.001) (12) to 4.19 (p=0.02) (10). Conversely, other investigations have described higher failure rates in the mandible, with HR between 2.03 (p&lt;0.05) (13) and 2.63 (p&lt;0.05) (14). There is also controversy regarding the influence of the anterior or posterior region on failure risk. Several studies have associated posterior implants with a higher risk of failure (10 , 12), possibly due to higher occlusal forces (15), lower bone density affecting primary stability (12), and increased plaque accumulation that may initiate or accelerate the progression of peri-implant disease. In contrast to these observations, a large retrospective study reported a higher risk of failure in anterior implants (OR=1.35; p=0.071) (11), potentially related to the frequent used of immediate implant placement in aesthetically demanding regions. Immediate implants may increase the risk of early failure by up to five-fold compared with delayed placement (5.1% vs. 1.1%, respectively; p=0.02) (16). Despite these observations, the present study did not identify a significantly higher failure rate in implants placed in pristine versus regenerated bone or between one and two-stage procedures. However, a higher failure rate was observed in implants placed simultaneously with transcrestal sinus elevation (16.7%), possibly due to reduced residual bone height and lower primary stability.

With respect to implant-abutment connection, most studies have reported no significant differences in failure rate, survival, or mechanical and biological complications between internal and external connections, although a significantly lower MBL has been consistently observed for internal connections. Meta-analyses have reported mean differences ranging from -0.25mm (p=0.01) (17) to -0.44mm (p&lt;0.00001) (18). In the present study, MBL was not evaluated; however, a significant lower failure rate was observed in internal connection implants compared with external connection implants (2.2% vs. 37.5%; p&lt;0.001), with no significant difference in survival time (55.21 months vs. 98.73 months, respectively; p=0.663). This finding may be explained by the role of increased MBL as a triggering factor for peri-implantitis, which may ultimately lead to implant failure.

The mixed Cox regression analysis identify three variables independently associated with implant failure: Postoperative implant infection, previous implant failure, and heavy smoking (&gt;20 cigarettes/day). Postoperative infection increased the risk of implant failure by 6.6-fold (p=0.004). When infection occurs before prosthetic loading, it may negatively interfere with osseointegration, thereby increasing the risk of early failure. Postoperative infections have been reported to affect approximately 1.7% of implants and 6.5% of patients, with a mean onset of 28 days (12 to 139 days) (19). In the present study, the infection rate was comparable (2.5% at the implant level), although the mean onset was earlier. The time between implant placement and infection diagnosis did not significantly influence the risk of failure (p=0.65). Preventive strategies should therefore focus on elimination of oral infection sources, maintenance of bacterial plaque levels below 20%, adequate control of systemic diseases, and appropriate use of PAT. In agreement with recent systematic reviews and meta-analyses (20 , 21), PAT was not associated with a lower failure rate in the present cohort. Nevertheless, when infection occurs during osseointegration, the probably of implant failure is high, which may justify selective use of PAT in high-risk procedures (22). On the other hand, when infection occurs after prosthetic connection, it is primarily due to two reasons: The development of peri-implantitis, or to hyperplastic fistulas and mucositis secondary, usually to the loosening of prosthetic components, as well as to food particles that are retained in the peri-implant sulcus that can occasionally cause mucosal abscesses (23). To avoid such complications, proper monitoring and follow-up of patients is important to identify these risk factors early.

Previous failure was also strongly associated with subsequent failure (HR=8.3; p&lt;0.001). A recent systematic review (24) reported a survival rate of 88.84% for replacement implants, with a gradual increase in failure risk over time (25). Repeated replacement implants appear to have progressively lower survival rates, highlighting the importance of careful case selection and risk stratification.

Finally, heavy smoking (&gt;20 cigarettes a day) showed a very strong association with implant failure. Smoking has been consistently associated with both early and late implant failure in a dose-dependent manner (26). Tobacco use directly and indirectly affects oral microflora, vascularisation, and tissue healing, thereby compromising osseointegration and peri-implant health (27). Although some authors suggest that consumption below 10 cigarettes/day may be relatively safer, heavy smokers exhibit more than double the risk of implant failure compared with light or moderate smokers (28).

The main strengths of this study include the large sample size, the exclusive use of a single implant system, and the multicentre design, which enhances external validity. However, several limitations should be acknowledged. Data on previous periodontal disease, MBL, medication use, and soft tissues parameters (i.e., tissue biotype and/or width of keratinised mucosa) were not available. In addition, operator variability and heterogeneity of regenerative procedures may have introduced confounding effects.

Future controlled prospective studies using standardised biomaterials and uniform selection criteria are warranted to further clarify the impact of surgical and patient-related factors on implant failure.

## Conclusions

With the limitations of this study, it can be concluded that the cumulative failure rate was low. However, particular caution should be exercised for implants placed in the maxilla and in the anterior region, as well as in patients with heavy smoking habits, as these factors significantly increase the risk of implant failure. More specifically, consumption of more than 20 cigarettes per day, postoperative infection, and replacement of a previously failed implant were independently associated with implant failure. Therefore, careful individualised treatment planning and strict control of modifiable risk factors are strongly recommended.

## Figures and Tables

**Table 1:** Descriptive study data.

Factors	Variable	Subvariable	N	%
Patient-related factors	Gender	Male	586	44.8
		Female	722	55.2
	Age (years)	<40	47	3.6
		40-65	801	61.2
		>65	460	35.2
	Smoking habit	No	930	71.1
		Yes	344	26.3
		Ex	34	2.6
	Cigarettes/day	0	961	73.5
		≤10	73	5.6
		11-20	257	19.6
		>20	17	1.3
Surgical technique and implant-related factors	Location	Anterior	215	16.4
		Posterior	1,093	83.6
	Arch	Maxilla	714	54.6
		Mandible	594	45.4
	DI connection	Internal	1,300	99.4
		External	8	0.6
	DI diameter	3.5mm	407	31.1
		4mm	703	53.8
		4.5mm	93	7.1
		5mm	104	8.0
	DI length	6mm	49	3.7
		8mm	337	25.8
		10mm	542	41.4
		12mm	328	25.1
		14mm	49	3.7
		16mm	3	0.2
	The DI was inserted in two phases (prior regeneration).	No	1,266	96.8
		Yes	42	3.2
	The DI was inserted in one phase (simultaneous regeneration).	No	1,076	82.3
		Yes	232	17.7
	Type of regenerative procedure performed	No	837	75.8
		Immediate DI	70	6.3
		Immediate DI + GBR	99	9.0
		Immediate DI + Transcrestal SFE	2	0.2
		GBR	58	5.3
		Transcrestal SFE	6	0.5
		Lateral SFE	26	2.4
		Socket shield	3	0.3
		Split crest	3	0.3
	Preventive antibiotic therapy	No	5	0.4
		Yes	1,303	99.6
	Postoperative infections	No	1,273	97.5
		Yes	32	2.5
	DI failure	No	1,276	97.6
		Yes	32	2.4
	Insertion of a repositioning DI	No	1,277	97.6
		Yes	31	2.4

%: Percentage. N: Frequency. DI: Dental implant. GBR: Guided bone regeneration. SFE: Sinus floor elevation.

**Table 2:** Factors related to the risk of implant failure.

Factors	Variable	Subvariable	N	%	p-Value
Patient-related factors	Gender	Male	17	2.9	0.338
		Female	15	2.1	
	Age (years)	<40	1	3.1	0.057
		40-65	26	81.3	
		>65	5	15.6	
	Smoking habit	No	17	1.8	0.022*
		Yes	15	4.4	
		Ex	0	0.0	
	Cigarettes/day	0	17	1.8	<0.001*
		≤10	0	0.0	
		11-20	7	2.7	
		>20	8	47.1	
Surgical technique and implant-related factors	Location	Anterior	10	22.0	0.022*
		Posterior	4.7	2.0	
	Arch	Maxilla	23	9.0	0.047*
		Mandible	3.2	1.5	
	DI connection	Internal	29	2.2	<0.001*
		External	3	37.5	
	DI diameter	3.5mm	7	1.7	0.235
		4mm	19	2.7	
		4.5mm	1	1.1	
		5mm	5	4.8	
	DI length	6mm	2	4.1	0.377
		8mm	5	1.5	
		10mm	12	2.2	
		12mm	10	3.0	
		14mm	3	6.1	
		16mm	0	0.0	
	The DI was inserted in two phases (prior regeneration).	No	32	2.5	0.297
		Yes	0	0.0	
	The DI was inserted in one phase (simultaneous regeneration).	No	26	2.4	0.879
		Yes	6	2.6	
	Type of regenerative procedure performed	No	21	2.5	0.094
		Immediate DI	1	1.4	
		Immediate DI + GBR	0	0.0	
		Immediate DI + Transcrestal SFE	0	0.0	
		GBR	4	6.9	
		Transcrestal SFE	1	16.7	
		Lateral SFE	0	0.0	
		Socket shield	0	0.0	
		Split crest	0	0.0	
	Preventive antibiotic therapy	No	0	0.0	0.723
		Yes	32	2.5	
	Postoperative infections	No	25	2.0	<0.001*
		Yes	7	21.9	
	Insertion of a repositioning DI	No	26	2.0	<0.001*
		Yes	6	19.4	

%: Percentage. N: Frequency. DI: Dental implant. GBR: Guided bone regeneration. SFE: Sinus floor elevation. *: Statistically significant.

**Table 3:** Average survival time of failed implants as a function of various variables.

Factors	Variable	Subvariable	Survival (months)	95% CI	p-value
Patient-related factors	Smoking habit	No	36.54	16.98-56.09	0.003*
		Yes	85.07	54.92-115.23	
	Cigarettes/day	0	36.54	16.99-56.09	<0.001*
		11–20	26.09	6.88-45.31	
		>20	136.68	136.16-137.21	
Surgical technique and implant-related factors	Location	Anterior	83.70	43.77-123.63	0.058
		Posterior	48.20	27.91-68.48	
	Arch	Maxilla	58.99	34.77-83.22	0.579
		Mandible	60.05	28.86-91.24	
	DI connection	Internal	55.21	34.58-75.85	0.663
		External	98.73	83.05-114.41	
	Postoperative infections	No	59.84	38.89-80.79	0.430
		Yes	57.34	7.71-106.98	
	Insertion of a repositioning DI	No	67.54	45.36-89.72	0.025*
		Yes	23.57	4.43-42.71	

%: Percentage. N: Frequency. DI: Dental implant. CI: Confidence interval. *: Statistically significant.

## Data Availability

Data available from the corresponding author on reasonable request.
